# Fast multiple-trait genome-wide association analysis for correlated longitudinal measurements

**DOI:** 10.1038/s41598-023-47555-1

**Published:** 2023-11-23

**Authors:** Gamal Abdel-Azim, Parth Patel, Shuwei Li, Shicheng Guo, Mary Helen Black

**Affiliations:** Janssen Res. & Dev. (Johnson & Johnson), Spring House, PA USA

**Keywords:** Computational models, Genome-wide association studies

## Abstract

Large-scale longitudinal biobank data can be leveraged to identify genetic variation contributing to human diseases progression and traits trajectories. While methods for genome-wide association studies (GWAS) of multiple correlated traits have been proposed, an efficient multiple-trait approach to model longitudinal phenotypes is not currently available. We developed GAMUT, a genome-wide association approach for multiple longitudinal traits. GAMUT employs a mixed-effects model to fit longitudinal outcomes where a fast algorithm for inversion by recursive partitioning of the random effects submatrix is introduced. To evaluate performance of the algorithms introduced and assess their statistical power and type I error, stochastic simulation was conducted. Consistent with our expectation, power was greater for cross-sectional (CS) than longitudinal (LT) effects, particularly with a diminishing LT/CS ratio. With a minimum minor allele count of 3 within genotype by time categories, observed type I error was roughly equal to theoretical genome-wide significance. Additionally, 28 blood-based biomarkers measured at 2 time points on participants of the UK Biobank were used to compare GAMUT against single-trait standard and longitudinal GWAS (including rate of change). Across all biomarkers, we observed 539 (CS) and 248 (LT) significant independent variants for the GAMUT method, and 513 (CS) and 30 (LT) for single-trait longitudinal GWAS, respectively. Only 37 variants were identified by modeling rates of change using standard GWAS.

## Introduction

Genome wide association studies have traditionally been carried out using cross-sectional data and one outcome at a time. With the advent of global biobanks and direct-to-consumer genomic testing, longitudinal health data recorded on diverse clinical aspects of each individual have opened the door for more statistically rigorous genome-wide association modeling. The availability of such data makes it possible to discover novel genetic variants associated with trajectories of trait changes or disease progression within individuals, as well as pleiotropic variants impacting multiple phenotypes.

Mixed models are the approach of choice for the analysis of longitudinal data because they account for variability between measurements across multiple time points on one phenotype as well as variability among measurements on multiple phenotypes^1, 2^. Mixed models are a commonly used approach for handling relatedness in conventional GWAS, e.g. as implemented with EMMA^3^, GEMMA^4^, and SAIGE^5^. While mixed models may also be used to model multiple-traits or longitudinal phenotypes, computational efficiency may be difficult to achieve for analysis of large-scale cohorts. This is due to the fact that mixed model approaches require the complex task of estimating variance components before setting up and solving a large system of equations, which requires the inversion of large matrices associated with the random components in the mixed model. Sikorska et al.^1, 6^ introduced computationally efficient approaches to utilize mixed models in longitudinal GWAS. Their approximate approaches were shown to be precise in estimating cross-sectional and longitudinal effects of each SNP, where cross-sectional effects are those comparable to conventional GWAS and longitudinal effects define how each SNP may impact the change in a trait over time. In an effort to simplify computations for longitudinal GWAS, researchers have often collapsed the multiple measurements into one rate of change between the earliest and the latest measurement of each individual. The rates of change were then fed into conventional GWAS workflows where associations with each phenotype were investigated independently. The collapsing approach was historically discussed and found to be theoretically problematic due to loss of information^7, 8^.

When individuals are assessed for multiple correlated phenotypes, a multivariate approach to jointly evaluate those phenotypes was shown to be more statistically rigorous^9, 10^. In addition, the availability of repeated measurements of the same phenotype on each individual affords an opportunity to assess genetic effects on changes in disease traits or biomarkers over time. Despite the advantages longitudinal and multiple-trait approaches each provides for identifying genetic determinants of disease and disease progression, implementation of such methods is also known to be computationally burdensome.

In the current study, we extend the mixed model approach, GALLOP^1^, for longitudinal phenotypes to a multiple-trait setting. The approach efficiently models multiple correlated phenotypes as well as multiple measurements recorded through time. To evaluate the computational efficiency of the approach as well as its ability to identify variant associations what would otherwise be missed with a conventional GWAS framework, 28 quantitative traits with repeated measures in up to 16,622 UK Biobank participants were assessed. Methods to obtain the inverse of large matrices are also introduced in an effort to maximize the computational efficiency of multiple-trait longitudinal GWAS.

## Material and methods

In standard GWAS, we test the association of each variant with the risk to a disease or to an extreme value of a quantitative trait, such as high cholesterol. In longitudinal GWAS, we test the association of the genetic variant with the progression of the disease or with the change in a quantitative biomarker for the disease over time. Single-trait longitudinal GWAS fits genetic variants one-by-one in the mixed model (1) which includes both the genetic effect of the variant and its interaction with the time interval from a baseline. In addition, the model may include time-dependent covariates, such as age and weight, and time-independent covariates such as sex. The following equation models a single phenotype, ${Y}_{ij}$, for individual $i$ at time point $j$,

1 $${Y}_{ij}=\mu +{at}_{j}+c{s}_{i}+l{s}_{i}{t}_{j}+\sum_{\tau =1}^{\mathrm{\rm T}}{\alpha }_{\tau }{Cov}_{ij\tau }+\sum_{\gamma =1}^{\Gamma }{\beta }_{\gamma }{Cov}_{i\gamma }+{g}_{i0}+{g}_{i1}{t}_{j}+{\varepsilon }_{ij},$$

where $a$ is a common effect of time point ${t}_{j}$, $c$ and $l$ are the genetic variant cross-sectional and longitudinal effects with ${s}_{i}$ representing the genotype dosage of individual $i$, ${\alpha }_{\tau }$ are $\mathrm{\rm T}$ time-dependent covariates, ${\beta }_{\gamma }$ are $\Gamma$ time-independent covariates, and ${g}_{i0} \mathrm{\ and\ } {g}_{i1}$ are individual random intercept and slope effects. Further, ${Cov}_{ij\tau }$ is the value of covariate $\tau$ associated with individual $i$ at time point $j$, such as age and BMI; and ${Cov}_{i\gamma }$ is the value of covariate $\gamma$ associated with all observations on individual $i$, such as genetic sex.

Following from a standard GWAS of a single longitudinal phenotype, multiple-trait longitudinal GWAS have been explored and shown to improve statistical power for discovery. In this type of analysis, multiple correlated traits are jointly modeled and missing records are implicitly inferred based on the covariance between traits. Multiple-trait longitudinal GWAS is typically performed in 3 steps: estimating variance components, then constructing the components of the mixed-model equations, and performing the association analysis. First, genetic and environmental variances and covariances are estimated once using a null model without genotypes, and then these are utilized in a transformed version of Henderson’s mixed model equations that are used with minor modifications for the hypothesis testing of each variant separately.

Following the multiple-trait evaluation framework outlined in Ref.^10^, both the single- and multiple-trait mixed models can be written as in (2) and solutions are obtained by solving the linear system in (3). The difference between the single- and multiple-trait models is in the specification of the fixed and random incidence matrices, **X** and **Z**, and the genetic and residual variance matrices, **G** and **R**, respectively. Starting by modeling a single trait using the following mixed model,

2 $${\varvec{y}}={\varvec{X}}{\varvec{b}}+{\varvec{Z}}{\varvec{u}}+{\varvec{e}},$$

where ${\varvec{b}}$ and ${\varvec{u}}$ are vectors of fixed and random effects, respectively, and ${\varvec{e}}$ is a vector of random residuals. With ${\varvec{u}} \sim MVN\left(\varvec{0}, {\varvec{G}}\right) \mathrm{and }\, {\varvec{e}} \sim MVN(\varvec{0}, {\varvec{R}})$, estimates of ${\varvec{b}}$ and predictions of ${\varvec{u}}$ are obtained by solving the following system of mixed model equations,

3 $$\left[\begin{array}{cc}{{\varvec{X}}}^{\prime}{{\varvec{R}}}^{-1}{\varvec{X}}& {{\varvec{X}}}^{\prime}{{\varvec{R}}}^{-1}{\varvec{Z}}\\ {{\varvec{Z}}}^{\prime}{{\varvec{R}}}^{-1}{\varvec{X}}& {{\varvec{Z}}}^{\prime}{{\varvec{R}}}^{-1}{\varvec{Z}}+{{\varvec{G}}}^{-1}\end{array}\right]\left[\begin{array}{c}{\varvec{b}}\\ {\varvec{u}}\end{array}\right]=\left[\begin{array}{c}{{\varvec{X}}}^{\prime}{{\varvec{R}}}^{-1}{\varvec{y}}\\ {\varvec{Z}}^{\prime}{{\varvec{R}}}^{-1} {\varvec{y}}\end{array}\right].$$

To accommodate $K$ traits, the following changes were made to the components of (3). $${{\varvec{y}}}^{\boldsymbol{^{\prime}}}={\left[ \begin{array}{cccc}{{\varvec{y}}}_{1}^{\prime}& {{\varvec{y}}}_{2}^{\prime}& \dots & {{\varvec{y}}}_{K}^{\prime}\end{array}\right]}^{\prime}$$, where each ${{\varvec{y}}}_{k}$ for $k\in \left\{1, 2, \dots , K\right\},$ is an *n*-dimensional vector of phenotypes and *n* is the number of measurements per trait. $${\varvec{X}}=\left[\begin{array}{cccc}{{\varvec{X}}}_{1}& 0& 0& 0\\ 0& {{\varvec{X}}}_{2}& 0& 0\\ 0& 0& \ddots & 0\\ 0& 0& 0& {{\varvec{X}}}_{K}\end{array}\right]$$, which is sometimes denoted as ${\varvec{I}}\otimes {{\varvec{X}}}_{k}$, where ${{\varvec{X}}}_{k}$ is the fixed effects incidence matrix of the ${k}^{th}$ trait, ${\varvec{I}}$ is an identity matrix of order $K$, and the ⊗ operator defines the Kronecker product. Each ${{\varvec{X}}}_{{\varvec{k}}}$ is an *n* × *p*_*k*_ matrix with different numbers of columns, *p*, when traits have different covariates or fixed effects; we may also set missing measurements to zero. The number of rows in ${{\varvec{X}}}_{k}$ is *n*, the maximum number of records in any of the *K* traits. $${\varvec{Z}}=\left[\begin{array}{cccc}{{\varvec{Z}}}_{1}& 0& 0& 0\\ 0& {{\varvec{Z}}}_{2}& 0& 0\\ 0& 0& \ddots & 0\\ 0& 0& 0& {{\varvec{Z}}}_{K}\end{array}\right]$$, where each ${{\varvec{Z}}}_{k}$, corresponding with the ${k}^{th}$ trait, is a block diagonal matrix with *q*_*i*_ × 2 blocks where *q*_*i*_ is the number of time points of individual $i,$ and $\sum_{i}{q}_{i}=n$, the number of records per trait. This makes each ${{\varvec{Z}}}_{k}$ a matrix with dimensions n × 2N, where N is the number of individuals in the study. Each block is constructed with 1’s in the first column to adjust for individual-level cross sectional random effects, and time intervals in the second column to adjust for individual-level longitudinal random slopes. Each value in the second column is the difference in time between baseline and every subsequent observation for the individual. Thus, the top time interval, corresponding to the baseline observation, is always 0. Because the number of rows of each block matches the number of time points with any data available on the individual, rows of 0’s are inserted if missing measurements exist for one or more, but not all, traits.

Matrices **G** and **R** are large square matrices of orders $2NK$ and $nK$, respectively. Instead of directly inverting them, we employed the following identity of the inverse of the Kronecker product,

4 $$\left( {{\varvec{A}} \otimes {\varvec{B}}} \right)^{ - 1} = {\varvec{A}}^{ - 1} \otimes {\varvec{B}}^{ - 1} .$$

**Figure 1:**
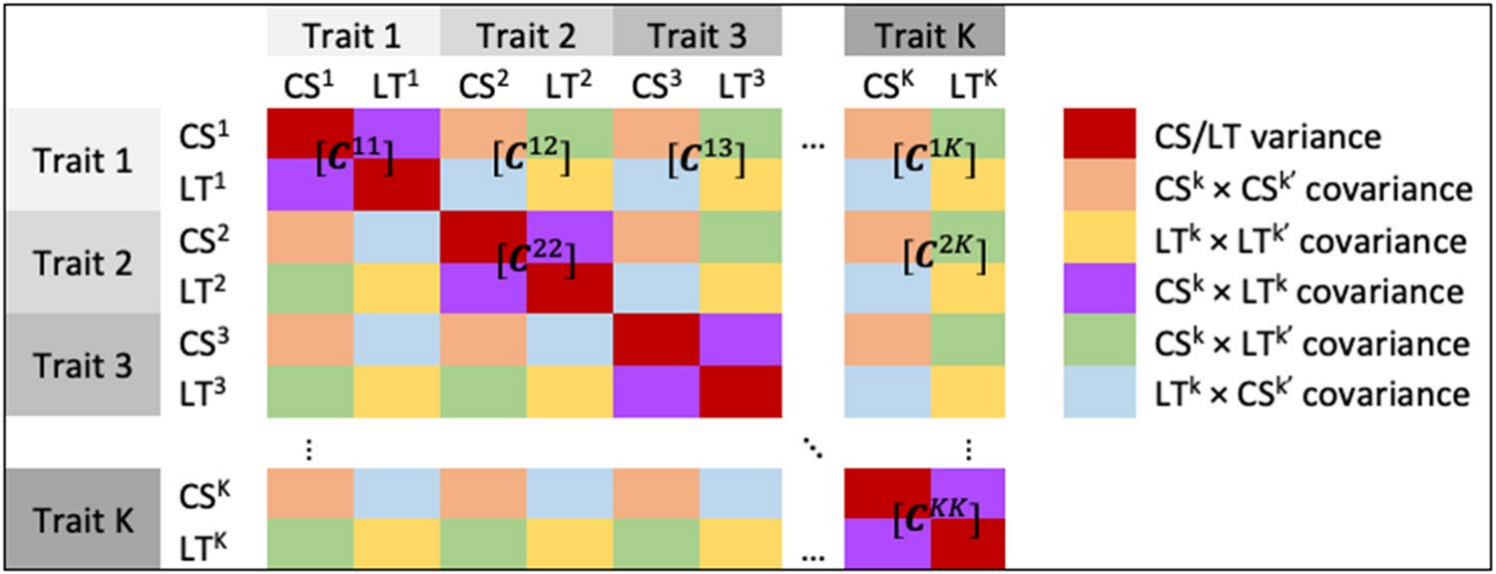
Inverse of the genetic variance and covariance matrix among $K$ traits. Each ${{\varvec{C}}}^{kk^{\prime}}$ submatrix corresponds to the ${(k{k}^{\prime})}^{th}$ 2 × 2 block of the pairwise covariance between cross-sectional and longitudinal effects of traits $k$ and $k^{\prime}$.

${{\varvec{R}}}^{-1}$ = ${{\varvec{E}}}^{-1}\otimes {{\varvec{I}}}_{n}$, where ${\varvec{E}}$ is a $K\times K$ residual covariance matrix between traits and ${{\varvec{I}}}_{n}$ is an identity matrix with order *n*. $${{\varvec{G}}}^{-1}=\left[\begin{array}{cccc}{{\varvec{I}}}_{N}\otimes {{\varvec{C}}}^{11}& {{\varvec{I}}}_{N}\otimes {{\varvec{C}}}^{12}& \cdots & {{\varvec{I}}}_{N}\otimes {{\varvec{C}}}^{1K}\\ & {{\varvec{I}}}_{N}\otimes {{\varvec{C}}}^{22}& \cdots & {{\varvec{I}}}_{N}\otimes {{\varvec{C}}}^{2K}\\ & & \ddots & \\ & & & {{\varvec{I}}}_{N}\otimes {{\varvec{C}}}^{KK}\end{array}\right]$$, where ${{\varvec{I}}}_{N}$ is an identity matrix or order N, ${{\varvec{C}}}^{kk^{\prime}}$ is the ${(kk^{\prime})}^{th}$ 2 × 2 covariance block of ${{\varvec{C}}}^{-1}$, and ${\varvec{C}}$ is the genetic covariance matrix between cross-sectional (CS) and longitudinal (LT) effects of all traits (Fig. 1). Matrices ${\varvec{C}}$ and ${\varvec{E}}$ were estimated in pairwise fashion for every 2 traits, generating $K\times (K-1)/2$ bi-trait analyses for variance components estimation.

Computationally efficient average information restricted maximum likelihood (AI-REML^11, 12^) was utilized in estimating variance components, in which a single covariance between effects of every 2 traits but $(K-1)$ variances for effects of each trait was generated.

### Inverting Z’R^–1^Z + G^–1^ by recursive partitioning

To simplify computations for the single-trait case, Sikorska et al.^1^ used eigen decomposition of a block diagonal matrix to transform the linear system in (3) so that the large lower right submatrix ${{\varvec{Z}}}^{\prime}{{\varvec{R}}}^{-1}{\varvec{Z}}+{{\varvec{G}}}^{-1}$ becomes an identity matrix. The decomposition is not suitable for the multiple-trait case because ${{\varvec{Z}}}^{\prime}{{\varvec{R}}}^{-1}{\varvec{Z}}+{{\varvec{G}}}^{-1}$ is no longer a block diagonal matrix. We arrived at a similar system transformed by pre-multiplying the system in (3) by $$\left[\begin{array}{cc}{\varvec{I}}& 0\\ 0& {\left[{{\varvec{Z}}}^{\prime}{{\varvec{R}}}^{-1}{\varvec{Z}}+{{\varvec{G}}}^{-1}\right]}^{-1}\end{array}\right]$$, which is not trivial to construct. An approach for inversion by recursive partitioning was developed so that the matrix is partitioned recursively along the block-diagonal submatrices and the inverse is carried out more swiftly. For any matrix $${\varvec{M}}=\left[\begin{array}{cc}{\varvec{A}}& {\varvec{B}}\\ {\varvec{C}}& {\varvec{D}}\end{array}\right]$$ with any 4 block-diagonal submatrices (denoted by ***A***, ***B***, ***C***, and ***D***), where **A** and **D** are square submatrices, the inverse is obtained using the identity,

5 $${{\varvec{M}}}^{-1}=\boldsymbol{ }\left[\begin{array}{cc}{({\varvec{A}}-{\varvec{B}}{{\varvec{D}}}^{-1}{\varvec{C}})}^{-1}& -{\left({\varvec{A}}-{\varvec{B}}{{\varvec{D}}}^{-1}{\varvec{C}}\right)}^{-1}({\varvec{B}}{{\varvec{D}}}^{-1})\\ {-({\varvec{D}}}^{-1}{\varvec{C}}){({\varvec{A}}-{\varvec{B}}{{\varvec{D}}}^{-1}{\varvec{C}})}^{-1}& {{\varvec{D}}}^{-1}+({{\varvec{D}}}^{-1}{\varvec{C}}){\left({\varvec{A}}-{\varvec{B}}{{\varvec{D}}}^{-1}{\varvec{C}}\right)}^{-1}({\varvec{B}}{{\varvec{D}}}^{-1})\end{array}\right].$$

**Figure 2:**
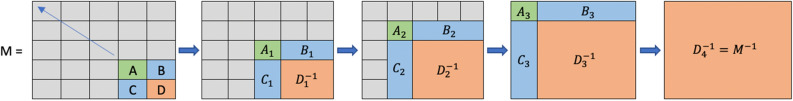
Schematic for inversion by recursive partitioning where the inverse of the lower-right corner of 4 submatrices is integrated in the next round as the inverse of the greater **D** (or **D**_1_), etc. Note that in each round, e.g. $\rho ,$ ${{\varvec{D}}}_{\rho }^{-1}$ replaced ${{\varvec{D}}}_{\rho }$ because the ${{\varvec{D}}}_{{\varvec{\rho}}}$ submatrix itself was not needed in round $\rho +1$.

For more than 4 blocks, the same identity in (5) were applied recursively while moving one row and column of submatrices each round. The inverse of the current round was stored to be used as the inverse of the growing submatrix **D** in the next round. As shown in Fig. 2, we started by inverting the lower-right corner of the 4 submatrices, **A**, **B**, **C**, and **D** at round 0 and used that as the inverse of the larger **D** submatrix at round 1, i.e. ${{\varvec{D}}}_{1}^{-1}$. The process continued until the entire matrix was inverted. To save computing storage, the inverse obtained in the current round substituted the **D** submatrix of the next round; note that the **D** matrix itself was not needed in the next round.

For the current multiple-trait model, the inverse was obtained using our proposed recursive partitioning in less computing time and resources than with other direct sparse inverse techniques (see “Results”) for the following reasons. First, indexes of the nonzero elements in the matrix and its inverse were the same, which significantly improved the efficiency of sparse storage. Second, the block diagonal structure of the 4 algebraic combinations in (5) is preserved throughout the recursive process, including intermediate steps, which makes it necessary to invert only 2 × 2 blocks within each submatrix combination. Thus, because $({\varvec{A}}-{\varvec{B}}{{\varvec{D}}}^{-1}{\varvec{C}})$ is block diagonal at any round, its inverse is obtained by inverting the diagonal blocks within this submatrix. Finally, the matrix and its inverse are symmetric and only one off-diagonal combination needs to be computed, i.e., only one of ${\left({\varvec{A}}-{\varvec{B}}{{\varvec{D}}}^{-1}{\varvec{C}}\right)}^{-1}\left({\varvec{B}}{{\varvec{D}}}^{-1}\right)$ or ${({\varvec{D}}}^{-1}{\varvec{C}}){({\varvec{A}}-{\varvec{B}}{{\varvec{D}}}^{-1}{\varvec{C}})}^{-1}$ is needed.

### Association analysis

Association analysis is then performed in a process slightly different from ref.^1^. In principle, the linear system in (3) was solved for each variant after including the equations for the variant as the first set of equations, i.e. bordering the system. The inverse of ${{\varvec{Z}}}^{\prime}{{\varvec{R}}}^{-1}{\varvec{Z}}+{{\varvec{G}}}^{-1}$ was calculated once using the recursive approach described above and then pre-multiplied by the linear system (3) to transform it to an equivalent system with an identity matrix in place of the large submatrix, ${{\varvec{Z}}}^{\prime}{{\varvec{R}}}^{-1}{\varvec{Z}}+{{\varvec{G}}}^{-1}$, as shown in (6), where **B** = ${{[{\varvec{Z}}}^{\prime}{{\varvec{R}}}^{-1}{\varvec{Z}}+{{\varvec{G}}}^{-1})}^{-1}$. The equivalent system is solved and the solutions vector, $$\left[\begin{array}{c}\widehat{{\varvec{b}}}\\ \widehat{{\varvec{u}}}\end{array}\right]$$ is stored. Because of the identity matrix in (6), inverting the left-hand side by partitioning was fast.

6 $$\left[\begin{array}{cc}{\varvec{I}}& 0\\ 0& {{[{\varvec{Z}}}^{\prime}{{\varvec{R}}}^{-1}{\varvec{Z}}+{{\varvec{G}}}^{-1}]}^{-1}\end{array}\right]\left[\begin{array}{cc}{{\varvec{X}}}^{\prime}{{\varvec{R}}}^{-1}{\varvec{X}}& {{\varvec{X}}}^{\prime}{{\varvec{R}}}^{-1}{\varvec{Z}}\\ {{\varvec{Z}}}^{\prime}{{\varvec{R}}}^{-1}{\varvec{X}}& {{\varvec{Z}}}^{\prime}{{\varvec{R}}}^{-1}{\varvec{Z}}+{{\varvec{G}}}^{-1}\end{array}\right]\left[\begin{array}{c}{\varvec{b}}\\ {\varvec{u}}\end{array}\right]=\left[\begin{array}{cc}{{\varvec{X}}}^{\prime}{{\varvec{R}}}^{-1}{\varvec{X}}& {{\varvec{X}}}^{\prime}{{\varvec{R}}}^{-1}{\varvec{Z}}\\ {{\varvec{B}}({\varvec{Z}}}^{\prime}{{\varvec{R}}}^{-1}{\varvec{X}})& {\varvec{I}}\end{array}\right]\left[\begin{array}{c}{\varvec{b}}\\ {\varvec{u}}\end{array}\right]=\left[\begin{array}{c}{{\varvec{X}}}^{\prime}{{\varvec{R}}}^{-1}{\varvec{y}}\\ {{\varvec{B}}({\varvec{Z}}}^{\boldsymbol{^{\prime}}}{{\varvec{R}}}^{-1}\boldsymbol{ }{\varvec{y}})\end{array}\right].$$

If the non-symmetric left-hand side matrix of the system in (6) was denoted by $$\left[\begin{array}{cc}{{\varvec{M}}}_{11}& {{\varvec{M}}}_{12}\\ {{\varvec{M}}}_{21}& {\varvec{I}}\end{array}\right]$$, and the right-hand side by $$\left[\begin{array}{c}{{\varvec{r}}}_{1}\\ {{\varvec{r}}}_{2}\end{array}\right]$$ then $\widehat{{\varvec{b}}}={\left({{\varvec{M}}}_{11}- {{\varvec{M}}}_{12}{{\varvec{M}}}_{21}\right)}^{-1}({{\varvec{r}}}_{1}-{{\varvec{M}}}_{12}{{\varvec{r}}}_{2})$ and $\widehat{{\varvec{u}}}={{\varvec{r}}}_{2}-{{\varvec{M}}}_{21}\widehat{{\varvec{b}}}.$ Note that inverting $\left({{\varvec{M}}}_{11}- {{\varvec{M}}}_{12}{{\varvec{M}}}_{21}\right)$, while a dense matrix, is still trivial because of its small order that is equivalent to the number of traits multiplied by the number fixed-factor equations.

Let us now construct and solve a system of equations with one SNP added to the null model, bordering the system of Eq. (6) as shown below in (7),

7 $$\left[\begin{array}{c|cc}{{\varvec{W}}}^{\prime}{{\varvec{R}}}^{-1}{\varvec{W}}& {{\varvec{W}}}^{\prime}{{\varvec{R}}}^{-1}{\varvec{X}}& {{\varvec{W}}}^{\prime}{{\varvec{R}}}^{-1}{\varvec{Z}}\\ \hline {{\varvec{X}}}^{\prime}{{\varvec{R}}}^{-1}{\varvec{W}}& {{\varvec{X}}}^{\prime}{{\varvec{R}}}^{-1}{\varvec{X}}& {{\varvec{X}}}^{\prime}{{\varvec{R}}}^{-1}{\varvec{Z}}\\ {{\varvec{B}}({\varvec{Z}}}^{\prime}{{\varvec{R}}}^{-1}{\varvec{W}})& {{\varvec{B}}({\varvec{Z}}}^{\prime}{{\varvec{R}}}^{-1}{\varvec{X}})& {\varvec{I}}\end{array}\right]\left[\begin{array}{c}{{\varvec{b}}}^{SNP}\\ \hline {\varvec{b}}\\ {\varvec{u}}\end{array}\right]=\left[\begin{array}{c}{{\varvec{W}}}^{\prime}{{\varvec{R}}}^{-1}{\varvec{y}}\\ \hline {{\varvec{X}}}^{\prime}{{\varvec{R}}}^{-1}{\varvec{y}}\\ {{\varvec{B}}({\varvec{Z}}}^{\boldsymbol{^{\prime}}}{{\varvec{R}}}^{-1}\boldsymbol{ }{\varvec{y}})\end{array}\right].$$

The structure of the SNP equations for the multiple-trait longitudinal GWAS is a straightforward extension of the single-trait structure. The matrix **W** in (7) can be expressed as ${\varvec{I}}\otimes {{\varvec{W}}}_{{\varvec{k}}}$, where ${\varvec{I}}$ is an identity matrix of order K and ${{\varvec{W}}}_{{\varvec{k}}}$ is a $\sum_{i}{q}_{i}\times 2$ matrix of cross-sectional and longitudinal effects, corresponding to the ${k}^{th}$ trait. The first column in ${{\varvec{W}}}_{{\varvec{k}}}$ is simply the vector of genotypes with elements of individual $i$ repeated ${q}_{i}$ times, and the second column equals the first column multiplied by time.

The system (7) is solved only for the 2 K elements of ${{\varvec{b}}}^{SNP}$ and their standard errors are extracted from the diagonal elements of the coefficient matrix inverse. If (7) is expressed as

8 $$\left[\begin{array}{cc}{{\varvec{H}}}_{11}& {{\varvec{H}}}_{12}\\ {{\varvec{H}}}_{21}& {{\varvec{H}}}_{22}\end{array}\right]\left[\begin{array}{c}{{\varvec{b}}}^{SNP}\\{\varvec{\lambda}}\end{array}\right]=\left[\begin{array}{c}{{\varvec{J}}}_{1}\\ {{\varvec{J}}}_{2}\end{array}\right],$$

then following (5),

9 $${\widehat{{\varvec{b}}}}^{SNP}={\left({{\varvec{H}}}_{11}-{{\varvec{H}}}_{12}{{\varvec{H}}}_{22}^{-1}{{\varvec{H}}}_{21}\right)}^{-1}\left({{\varvec{J}}}_{1}-{{{\varvec{H}}}_{12}{{\varvec{H}}}_{22}^{-1}{\varvec{J}}}_{2}\right),$$

and the standard errors of ${\widehat{{\varvec{b}}}}^{SNP}$ are obtained as

10 $${\varvec{S}}{\varvec{E}}=\sqrt{[diag{\left({{\varvec{H}}}_{11}-{{\varvec{H}}}_{12}{{\varvec{H}}}_{22}^{-1}{{\varvec{H}}}_{21}\right)}^{-1}}].$$

Clearly Eqs. (9) and (10) include cumbersome expressions, particularly the inverse of the large ${{\varvec{H}}}_{22}$ matrix. However, following a similar approach to ref.^1^, ${{{\varvec{H}}}_{22}^{-1}\boldsymbol{ }{\varvec{J}}}_{2}$ is simply the solutions of the system (6) discussed earlier, which need to be calculated only once and stored. ${{\varvec{H}}}_{22}^{-1}{{\varvec{H}}}_{21}$ is again the solutions of (6) but with ${{\varvec{H}}}_{21}$ replacing the right-hand side. Therefore, the inverse of ${{\varvec{H}}}_{22}$ is never calculated explicitly. Finally, the p-values associated with the elements of ${\widehat{{\varvec{b}}}}^{SNP}$ are obtained as double the area under the standard normal density with integration limits $\left\{\left|\frac{{\widehat{{\varvec{b}}}}^{SNP}}{{\varvec{S}}{\varvec{E}}}\right|,\boldsymbol{ }\boldsymbol{\infty }\right\}$.

### Simulation to study power and type I error of multiple-trait longitudinal GWAS

For individual $i$, the cross-sectional component was simulated as ${u}_{i}=\sum_{r}{\beta }_{r}{g}_{ir}$ and the longitudinal component was simulated as ${v}_{ij}=\sum_{r}{\delta }_{r}{t}_{ij}{g}_{ir}$, where ${\beta }_{r}$ and ${\delta }_{r}$ are the cross-sectional and longitudinal effects, respectively, of SNP $r$; ${g}_{ir}$ is the genotype of individual $i$ for SNP $r$; and ${t}_{ij}$ is the *jth* time point of individual $i$. Only 2 time points were considered with intervals between 4 to 9 years from the baseline. The phenotype value of individual $i$ at time $j$ was constructed as ${\alpha }_{0}+{\alpha }_{1}{t}_{ij}+{\alpha }_{2}{Cov}_{i}+{u}_{i}+{v}_{ij}+{\varepsilon }_{ij}$, where ${\alpha }_{0}$, ${\alpha }_{1}$, ${\alpha }_{2}$, are overall mean and regression coefficients on scaled time, ${t}_{ij}$, and a time-independent covariate, ${Cov}_{i}$. Finally, ${\varepsilon }_{ij}$ is a random residual component corresponding to individual $i$ at time point $j$.

Three correlated traits were considered in the current simulation with 3 CS and 3 LT effects for each SNP. We use the vector $${{\varvec{a}}}_{r}^{\prime}=\left[\begin{array}{cccccc}{\beta }_{r1}& {\beta }_{r2}& {\beta }_{r3}& {\delta }_{r1}& {\delta }_{r2}& {\delta }_{r3}\end{array}\right]$$ to denote the effects of SNP $r$ on the 3 simulated traits, where ${\beta }_{rk}$ and ${\delta }_{rk}$ are cross-sectional and longitudinal genetic effects, respectively, of SNP $r$ on trait $k$, $k\in \left\{1, 2, 3\right\}$. Genetic effects in ${{\varvec{a}}}_{r}^{\prime}$ were sampled from a multivariate normal distribution, $MVN\left({\varvec{\mu}},{\varvec{\Sigma}}\right)$, where ${\varvec{\mu}}=E\left({{\varvec{a}}}_{r}\right)$ and ${\varvec{\Sigma}}=\mathrm{var}\left({{\varvec{a}}}_{r}\right)$. $E\left({\beta }_{rk}\right)$ was set to 10.0 and $E\left({\delta }_{rk}\right)$ = $E\left({\beta }_{rk}\right)/L$, where $L\ge 1$, is a scalar used to simulate longitudinal effects smaller than or equal to cross-sectional effects. With ${{\varvec{a}}}_{r}^{\prime}$ represented as $$\left[\begin{array}{cc}{{\varvec{\beta}}}_{r}^{\boldsymbol{^{\prime}}}& {{\varvec{\delta}}}_{r}^{\boldsymbol{^{\prime}}}\end{array}\right]$$, where $${{\varvec{\beta}}}_{r}^{\boldsymbol{^{\prime}}}=\left[\begin{array}{ccc}{\beta }_{r1}& {\beta }_{r2}& {\beta }_{r3}\end{array}\right]$$, and $${{\varvec{\delta}}}_{r}^{\boldsymbol{^{\prime}}}=[\begin{array}{ccc}{\delta }_{r1}& {\delta }_{r2}& {\delta }_{r3}\end{array}]$$, ${var({\varvec{\beta}}}_{r})$= $$\left[\begin{array}{ccc}10& 8& -7\\ 8& 12& -1\\ -7& -1& 18\end{array}\right]$$, ${var({\varvec{\delta}}}_{r})$ = ${var({\varvec{\beta}}}_{r})/L$, and ${covar({\varvec{\beta}}}_{r}, {{\varvec{\delta}}}_{r})$ = $$\left[\begin{array}{ccc}0.5& 0.4& -0.35\\ 0.4& 0.6& -0.05\\ -0.35& -0.05& 0.9\end{array}\right]$$. Finally, the residuals covariance matrix was simulated with equal values to those in the covariance matrix of the cross-sectional effects which resulted in a heritability value slightly above 50% at baseline. Heritability exceeded 50% in simulation due to the addition of the longitudinal genetic component.

In our simulation strategy, we chose to reduce the covariance between cross-sectional and longitudinal effects. We tested multiple ratios for the cross-sectional to longitudinal effects by setting the value of $L$ to 1, 5, 20, 50 or 100 to study its effect on power and type I error. Out of 10,000 SNP genotypes simulated for each individual, 100 causal SNPs were drawn at random. To simulate background genetic effects, infinitely small effects were drawn from $MVN({\varvec{\mu}}/1000,{\varvec{\Sigma}}/1000)$ for the non-causal SNPs. We studied the effect of sample sizes from 1,000 to 10,000 on the multiple trait longitudinal GWAS power and type I error. Only variants with an allele count ≥ 3 within each genotype by time category were tested, i.e. minimum allele frequency, of a vector constructed by multiplying available genotype dosages of each variant by time intervals, was required to exceed 3 divided by the number of individual samples. Further, allele frequencies were sampled from a beta distribution with shape parameters, $\alpha =2 \& \beta =10$. A minimum allele frequency threshold was set to 0.01 by adding 0.01 to the frequencies generated. Finally, genotype dosages {0, 1, 2} were randomly and independently sampled for each genetic variant with Hardy–Weinberg probabilities.

### Analysis of 28 blood biomarkers in the UK Biobank data

The UK Biobank is a prospective cohort study with rich genetic and health data from half a million participants. The UK Biobank study was carried out in accordance with relevant guidelines and regulations as approved by the NHS National Research Ethics Service (approval letter dated 17th June 2011, Ref 11/NW/0382). Participants in the study gave full informed signed consent.

First and second measurements of 28 blood biomarker traits of UK Biobank participants of European ancestry were evaluated for cross-sectional and longitudinal associations with imputed array genotypes. Phenotypes assessed were from UK Biobank field IDs (cf. Table 2): 30600, 30610,…, 30890; field IDs 30800 and 30820 were excluded due to low sample size (N ≤ 1009 participants). Sample size of individuals with 2 measurements for each field ID, of the remaining 28 biomarkers, ranged from N = 12,203 to 16,622. Participant age ranged 40 to 79 years and intervals between baseline and first repeat measurement ranged 2 to 6 years (median: 4 years). Imputed array genotypes from the UK Biobank were filtered retaining binary non-monomorphic SNPs with minor allele frequency greater than 1% and in Hardy–Weinberg equilibrium.

Phenotypes were transformed using rank-based inverse normal transformation and clinical and demographic covariates other than time intervals and principal components of ancestry were scaled to a mean of 0 and variance of 1. Traits were clustered into 4 clusters according to the magnitude and direction of the correlation between traits. Simple hierarchical clustering on the correlation matrix using the Ward’s method^13^ was performed. Cluster sizes ranged from 3 to 12 where traits within each cluster were analyzed jointly using GAMUT. For comparison, data were also analyzed using the single-trait approach, GALLOP^1^.

In addition to the first 20 principal components for ancestry, covariates included time, sex, year of birth, year of birth squared, year of birth by sex interaction, assessment center, and whether the individual was taking cholesterol-lowering medications at the time of blood sampling. Covariates differed across measurements and traits, for example, assessment centers and times were different between the first and second measurements, whereas cholesterol-lowering medications were considered only with cholesterol, LDL direct, and Apolipoprotein B. In addition to the statins list of medications, cholesterol-lowering drugs considered here included those that inhibit cholesterol absorption in the intestines and PCSK9 inhibitors for patients with heterozygous familial hypercholesterolemia (HeFH) and heart disease who may need more than statin.

To compare GALLOP and GAMUT with an approach that evaluates SNPs for their associations with rates of change, a single value per individual was derived as the difference between the two measurements divided by the time interval between measurements. The GWAS model for rates of change did not include time but included the baseline measurement as a scaled covariate. All other covariates were the same across the methods studied. Models for rates of change included those without both baseline and time, as well as an addition of both baseline and time.

### Triglycerides in the primary care UK Biobank data

To test the impact of comorbidity and the number of repeated measures on the longitudinal GWAS outcomes, triglycerides (TG) were extracted from the primary care data, referred to as general practice or GP data. TG measures on 8968 participants of European ancestry who were diagnosed with coronary artery disease (CAD) at any age using all available clinical history information were extracted from the UK Biobank GP data. The dataset included nearly 99,000 observations on 8968 individuals with 3 to 35 TG measures per participant over an average period of 6 years. A comparable group of 8587 individuals with 3–35 repeated measures per individual was randomly selected from the UK Biobank cohort for the purpose of comparison. The objective was to evaluate the reliability and benefit of controlling for diseases interfering with biomarker measures on the quality of longitudinal effects. With the onset of such diseases, trait trajectories might be perturbed in a way that impairs the detection of true genome-wide longitudinal signals. A single trait longitudinal GWAS was run on each dataset and the impact of comorbidity on the quality of estimates was reported. Mixed models for the two datasets were adjusted for cholesterol-lowering drugs as they might lower triglycerides as well.

## Results

### Power and type I error in simulation

**Figure 3:**
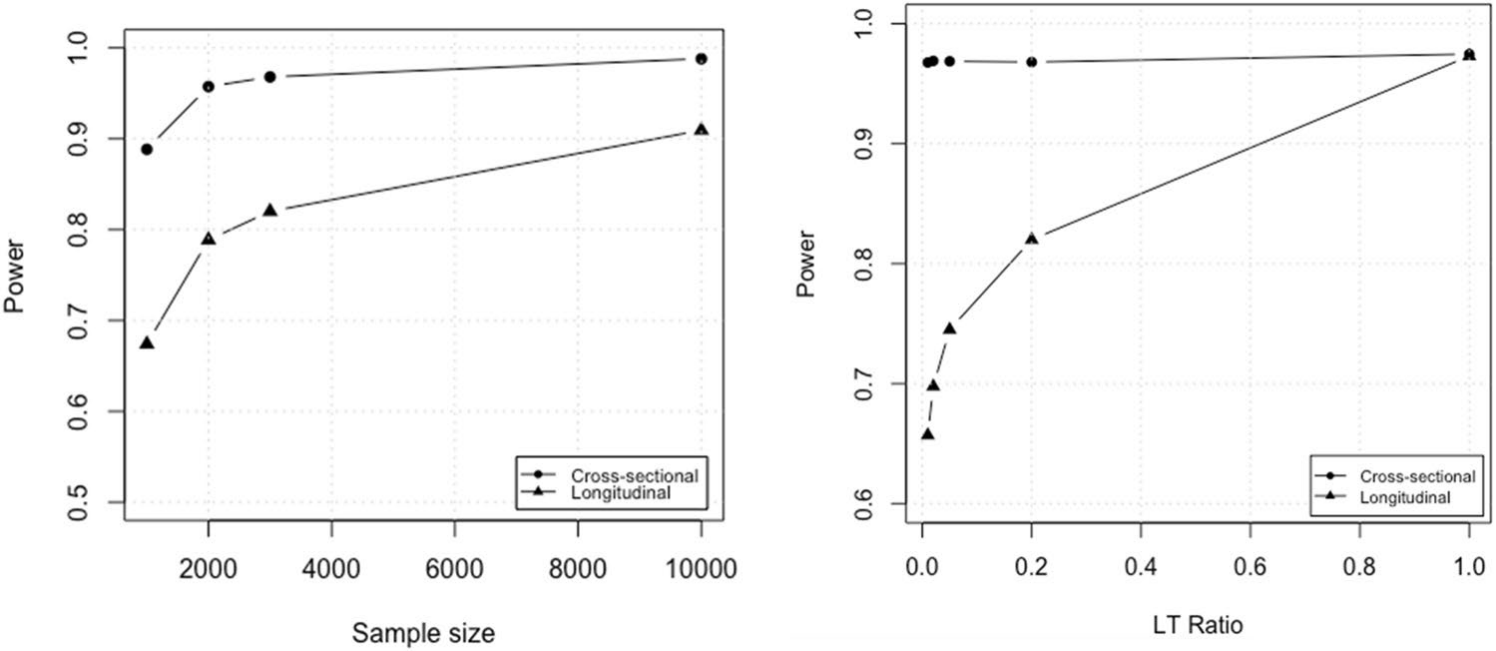
Statistical power of identifying causal variants using GAMUT. Powers are shown for 4 sample sizes in the left panel and 5 longitudinal to cross-sectional ratios in the right panel. Powers shown are calculated as averages of 3 phenotypes over 100 replicates per scenario. Power of detecting causal longitudinal effects was smaller than those of cross-sectional effects, particularly for relatively smaller longitudinal effects. Sample size simulations of the left panel were based on 1:5 LT to CS, ratios which resulted in a consistently lower longitudinal curve.

Four sample sizes of one, two, three and ten thousand individuals were simulated. In addition, 5 longitudinal to cross-sectional ratios of 1, 2, 5, 20, and 100% (LT ratio scenarios) were simulated and replicated 100 times. LT ratio scenarios were simulated with 3000 individuals each; and sample size scenarios were simulated with LT effects that were 20% of the CS effects (LT to CS ratio of 1:5). Data sets simulated were analyzed with GAMUT and GALLOP. As shown in Fig. 3, power to detect causal LT effects was consistently smaller than that for CS effects. In fact, only at a sample size of 10,000 did the power for LT analysis exceed that of CS (left panel of Fig. 3). This was due to the fact that LT effects were simulated to be much smaller than CS effects, which is likely to be the common scenario seen in real world data. The right panel of Fig. 3 further validates the impact of LT/CS ratio down to 1% on longitudinal GWAS power.

Bonferroni-corrected significance threshold was calculated as 0.05 divided by the total number of variants scanned. Type I error realized in the simulation was taken as the proportion of non-causal variants with p-values below the Bonferroni-corrected significance threshold. Table 1 shows average type I error for both CS and LT effects estimated by GAMUT. For sample size and LT ratio scenarios, type I error was sufficiently small and similar to genome-wide values.

**Table 1:** GAMUT average type I errors associated with sample-size and LT-ratio scenarios.

Scenario	Bonferroni-corrected threshold	Type I error (CS)	Type I error (LT)
Sample size	8.86E-06	1.16E-05 ± 8.80e-07	3.93E-05 ± 3.86e-06
LT Ratio	7.34E-06	7.85E-06 ± 6.12e-07	2.28E-05 ± 1.01e-06

Bonferroni-corrected significance threshold was obtained by dividing 0.05 by the total number of variants scanned and passed minor allele count of 3 within a genotype by time class. Type I error was sufficiently small. Sample size scenarios were based on a 1:5 LT to CS ratio, and LT ratio scenarios were based on a sample size of 3000 individuals.

**Figure 4:**
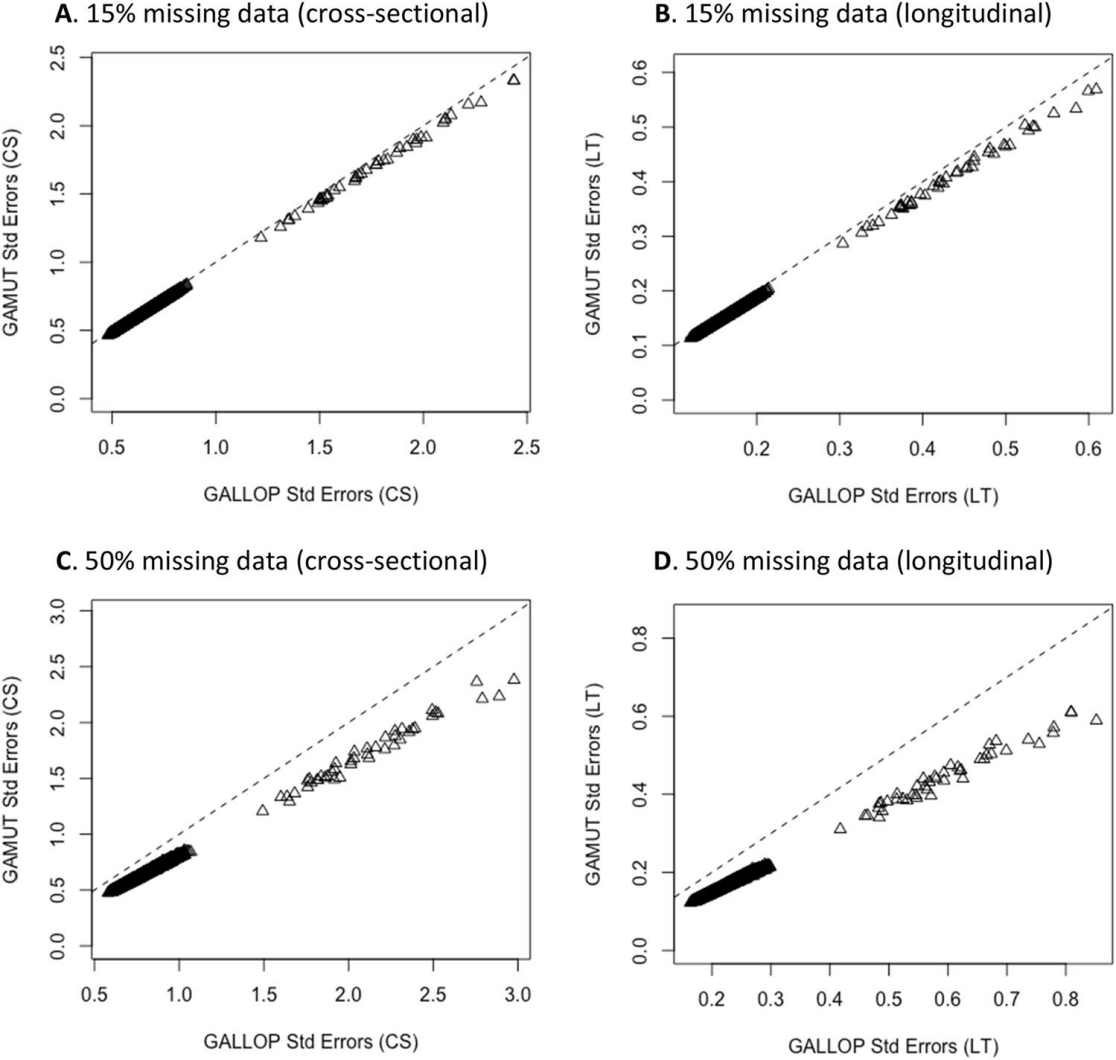
Standard errors of multiple-trait, GAMUT versus single-trait, GALLOP for two scenarios, one with 15% missing records (**A**,**B**) and another with 50% missing records (**C**,**D**) in 1 out of 3 simulated phenotypes. GAMUT consistently reduced standard errors of genetic variants scanned for the phenotype with missing records. Dotted line is the slope of GAMUT Std Errors on equivalent values.

To study statistical power for the multiple- versus single-trait approach, standard errors of all variants in two simulation runs were compared. In the first run, 15% of values were assigned to be randomly missing for one of the three phenotypes and in the second run, 50% randomly selected records were set to be missing for the same phenotype. Standard errors were consistently smaller with the multiple trait analyses, especially when a greater proportion of data was missing. In addition, Fig. 4 shows that GAMUT was particularly useful in testing LT effects as can be seen from their greater standard errors with GALLOP (Fig. 4B and D) relative to their corresponding CS effects (Figs. 4A and C). Results shown in Fig. 4 are only for the phenotype with missing records, phenotypes with non-missing data were not impacted.

**Figure 5:**
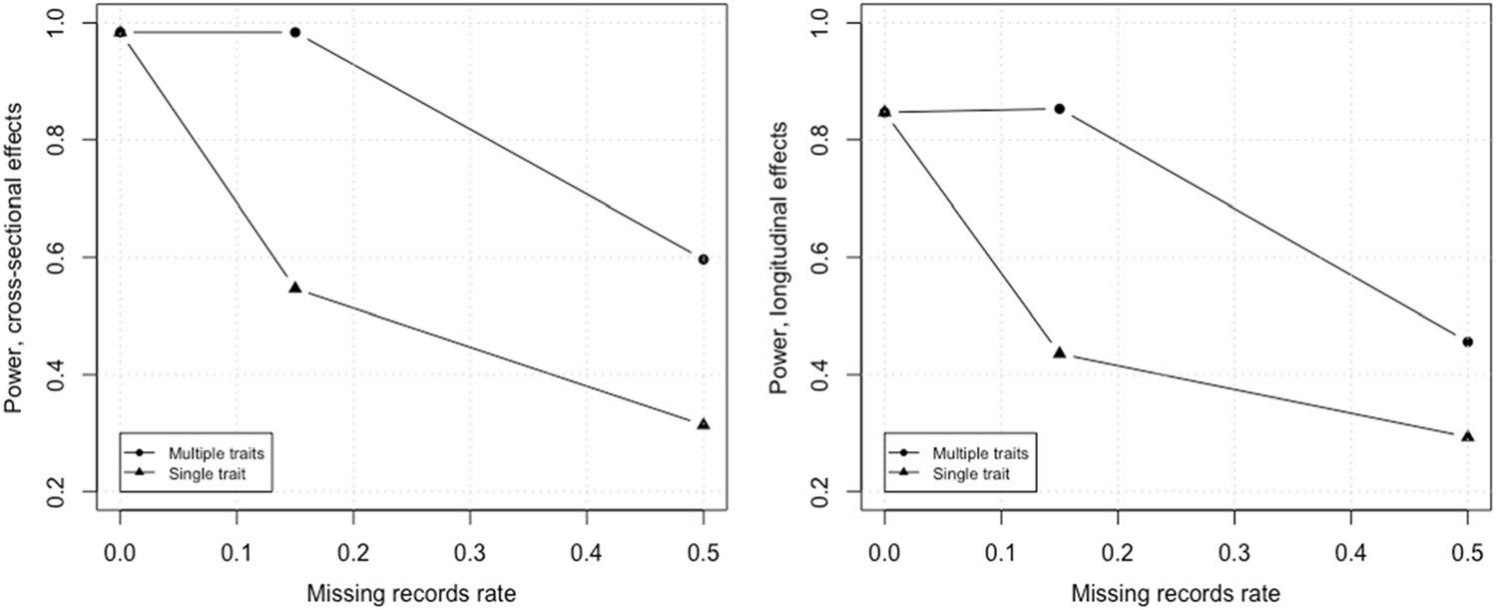
Cross-sectional and longitudinal statistical power estimates in simulated scenarios with 0, 15, and 50% of individuals missing. In the single-trait approach, missing individuals directly impacted sample size and significantly reduced power of the phenotype with missing data, relative to the multiple-trait approach which utilized the correlation between traits to compensate for the reduction in sample size. In the simulation, the sample size with no missing records was 3000 and the LT to CS ratio was set to 1:5.

Because standard errors were shown to be reduced for all variants, both causal and noncausal, power and type I errors associated with multiple- vs. single-trait approaches were studied in a scenario where 15 and 50% of individuals were missing for their baseline and second records. Figure 5 shows the multiple trait advantage when a proportion of individuals in data, 15 and 50%, were partially measured for one phenotype and completely measured for the other two. In the single-trait case this directly impacted the sample size for the partially measured phenotype because the analysis was run without the individuals with missing records. In the multiple-trait case, analysis was run with all individuals as long as they were measured for at least 1 of the 3 phenotypes, which boosted statistical power. Note that Fig. 5 shows the performance of the two approaches using the phenotype with missing records. Further, type I errors were shown to be controlled in the simulation as they ranged from 5.97e-06 to 1.82e-05 versus a theoretical average of 7.34e-06.

### Benchmarking GAMUT in UK Biobank data

Performance of GAMUT vs. other approaches in the context of real-world data is shown next. GAMUT results are compared with a single-trait longitudinal approach using GALLOP and a single-trait conventional GWAS on each phenotype rate of change using REGENIE^14^. Variants detected by the 3 approaches are reported in the following sections. Benchmarking data included 28 blood biomarkers in the UK Biobank, where all individuals with 2 measurements for at least one biomarker were utilized in the analyses.

#### System setup and association time

**Figure 6:**
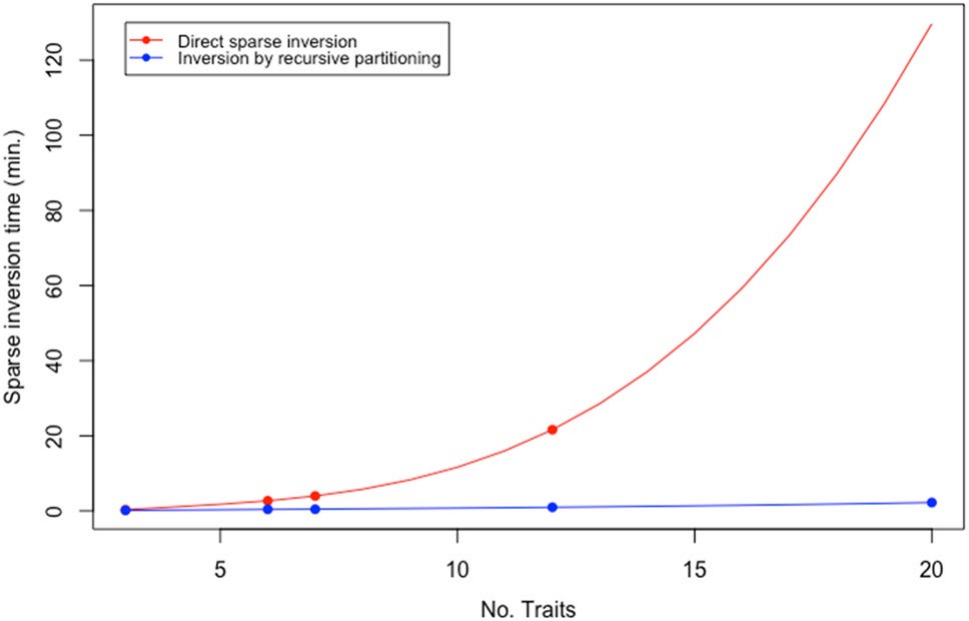
Actual time for direct sparse inversion and inversion by recursive partitioning of **Z**’**R**^–1^**Z** + **G**^-–1^. For 20 traits, runtime for direct sparse invasion was extrapolated to 130 min vs. 2.2 min of actual runtime for inversion by recursive partitioning. Direct inversion was highly exponential versus recursive inversion that was nearly linear in the number of traits.

Inversion by recursive partitioning was highly efficient and greatly reduced system setup time. For 12 traits, inversion by recursive partitioning of **Z**’**R**^–1^**Z** + **G**^–1^ took 59 s vs. 21.6 min using direct sparse inversion. For 20 traits, inversion by recursive partitioning took 2.2 min vs. extrapolated 130 min consumed by direct sparse inversion (Fig. 6); direct sparse inversion was not possible to carry out on the same machine. Inversion by recursive partitioning greatly improved the efficiency of setting up the mixed model system of equations.

**Figure 7:**
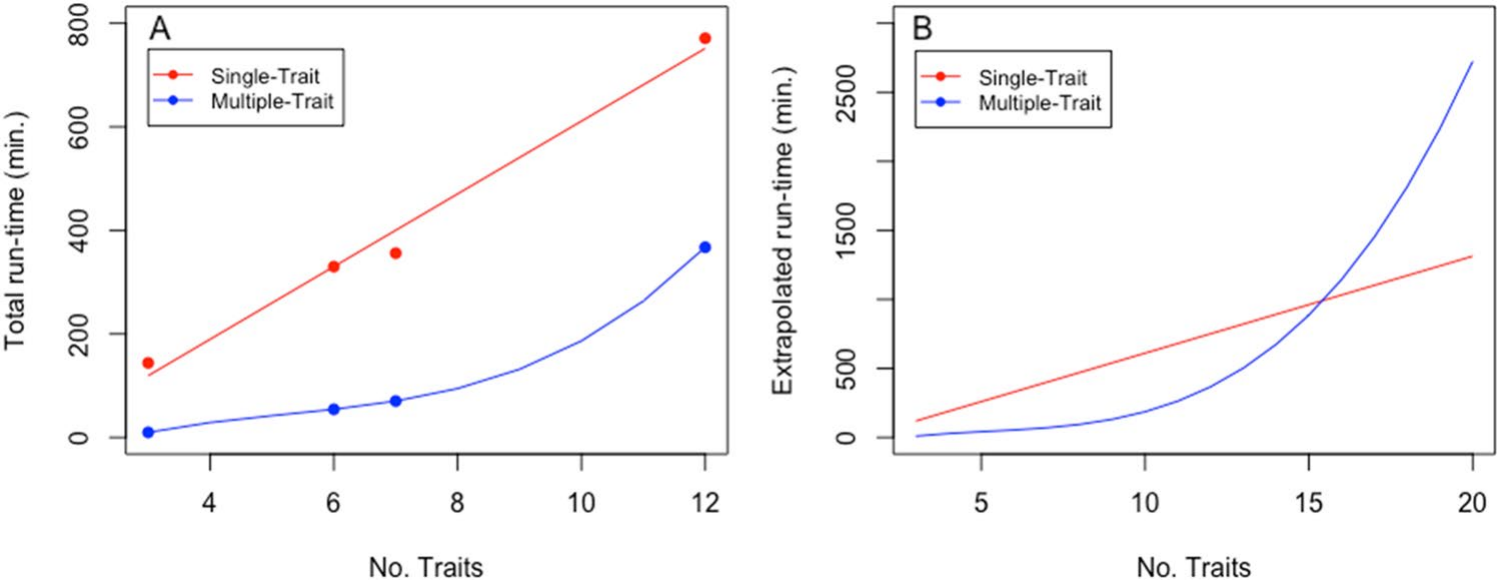
(**A**) Total system setup and association times for the single- and multiple-trait runs, modeled up to 12 traits in the largest cluster. Multiple-trait analyses were far more run-time efficient vs. single-trait, despite following an exponential curve. (**B**) extrapolated time up to 20 traits in analysis; multiple-trait cost exceeded that of single-trait after 16 traits.

For the 4 clusters of traits, GAMUT was more efficient than GALLOP (Fig. 7A). By extrapolation, the run-time cost for GAMUT exceeded the run-time cost of GALLOP only after 16 traits (Fig. 7B). Because traits were modeled independently for the single-trait approach and jointly for the multiple-trait approach, the run-time cost was linear for GALLOP and exponential for GAMUT. We developed and implemented our method in a high performance computing environment with 16 CPU cores and 32 GB of RAM for inversion and system setup and only 12 GB of RAM for scanning genetic variants. The differences in performance reported for the inversion by recursive partitioning vs. direct sparse inversion are large enough to be observed on any hardware.

#### Variance components estimation in blood biomarker data

**Figure 8:**
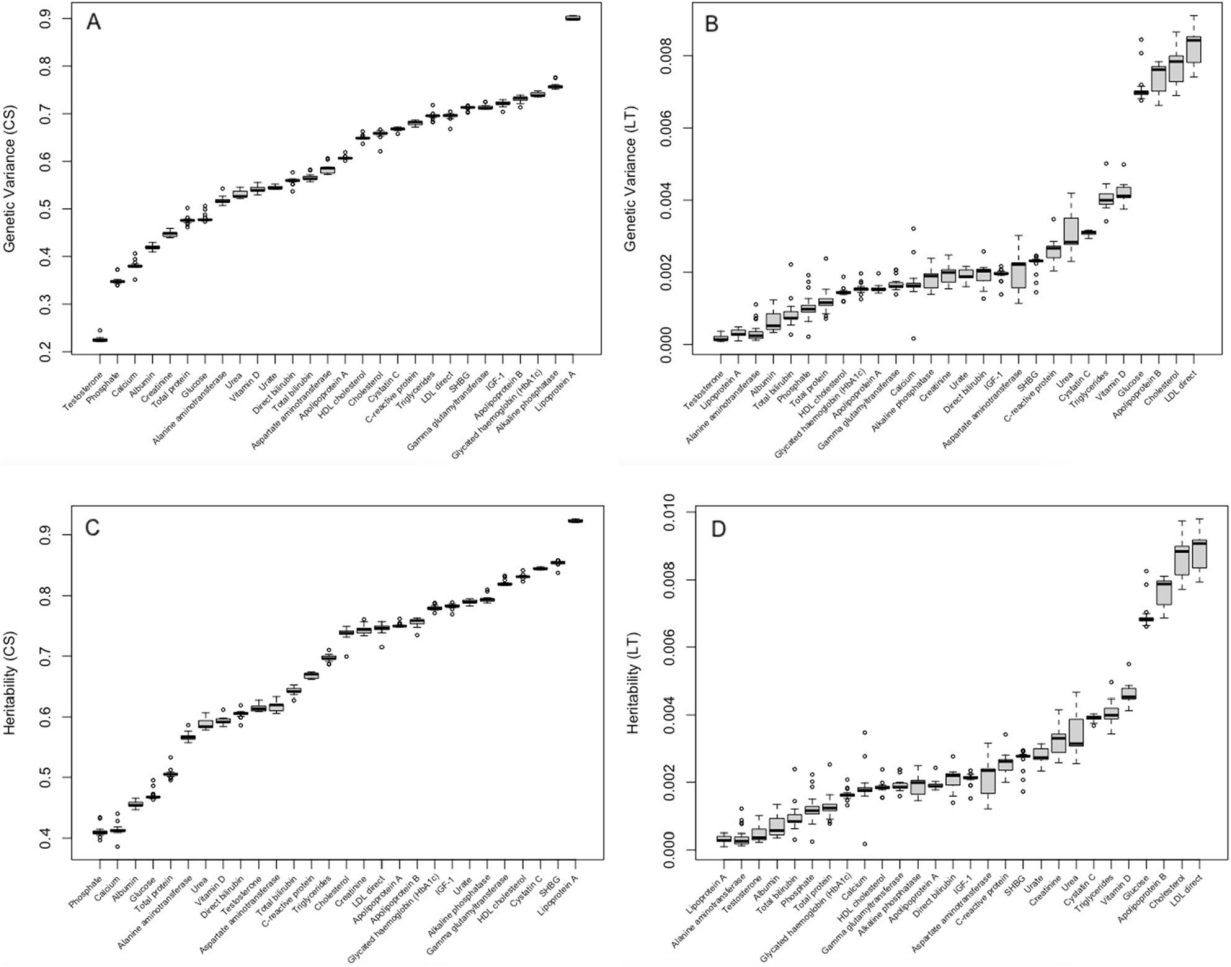
(**A**) Polygenic cross-sectional (CS) variances estimated between traits in a pairwise fashion where Lipoprotein A produced the highest variance and Testosterone showed the least genetic variance. (**B**) Longitudinal variances (LT) variances estimated alongside the CS variances using AI-REML. LDL direct, Cholesterol, Apolipoprotein B, and Glucose had the greatest variances and Testosterone had near-zero longitudinal variance. (**C**) Cross-sectional polygenic heritability estimates. (**D**) Longitudinal polygenic heritability estimates. CS polygenic variance and heritability estimates were generally much greater than those for LT, indicating that more cross-sectional associations are expected to be identified. Further, CS polygenic variance and heritability estimates were relatively more consistent compared with those for LT.

Before running GAMUT, a total of (28*27)/2 = 378 pair-wise variance component sets of estimates were obtained using AI-REML. Although the variance of each trait was estimated multiple times when paired with other traits, covariances estimated were unique. CS and LT variances of each biomarker trait in a genetic variance–covariance matrix of order 56 were averaged across all other traits. Pairwise covariances were unique. Residual variances and covariances between traits were similarly summarized, where variances were averaged while pairwise covariances were unique. Although variances were estimated multiple times, they were consistent, especially for the CS components, as the boxplots of Fig. 8A and B show. For all traits in the study, LT variances were significantly smaller than CS variances which should be reflected in the expected number of significant longitudinal associations to be found, i.e. reduced power to discover longitudinal associations, as was also validated by simulation in the current study. Cross-sectional and longitudinal heritability estimates were obtained as the proportion of total variance attributed to polygenic CS and LT variances, respectively (Fig. 8C and D). Cross-sectional heritability values shown in Fig. 8C were calculated as ${var}_{CS}/({var}_{CS}+{var}_{LT}+{var}_{Residual})$ and longitudinal heritability values plotted in Fig. 8D were calculated as ${var}_{LT}/({var}_{CS}+{var}_{LT}+{var}_{Residual})$. Note that this is a polygenic heritability obtained using the null model (3) without SNP effects and with a block diagonal covariance matrix, not a genetic relationship matrix (GRM).

**Figure 9:**
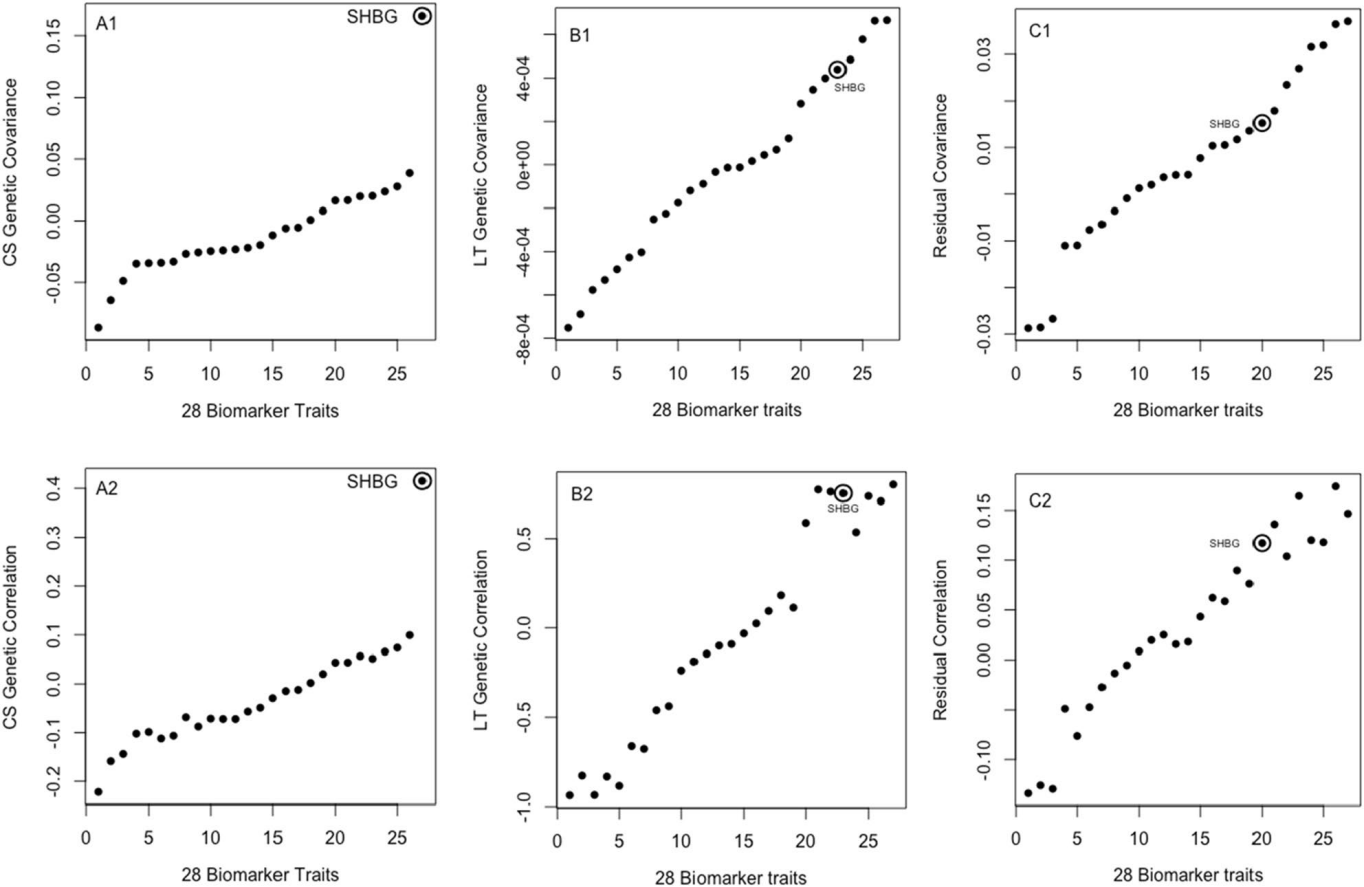
Polygenic and residual covariance and correlation between Testosterone and all other biomarker traits, sorted by covariance. (**A1**,**A2**) Cross sectional variance components estimates, showing SHBG (circled points on the scatter plot) as the trait with the strongest correlation. SHBG is a protein made by the liver and binds itself to sex hormones in both sexes. (**B1**,**B2**) Longitudinal variance component estimates, showing SHBG among the top correlated traits, indicating parallel progression at the genetic level between the two traits. (**C1**,**C2**) show the residual variance component estimates with high SHBG correlation that is not as strong as the genetic correlations.

The weakest variance components were estimated for Testosterone, measured in both sexes. Figure 9A1,A2,B1,B2,C1 and C2 show covariances and correlations between Testosterone and every other trait for the cross-sectional, longitudinal, and residual components. Despite the low variance components estimated for the trait, correlations were biologically sound as shown by the strong genetic correlation between Testosterone and sex hormone binding globulin (SHBG), a protein produced by the liver and attaches itself to sex hormones in both men and women.

**Figure 10:**
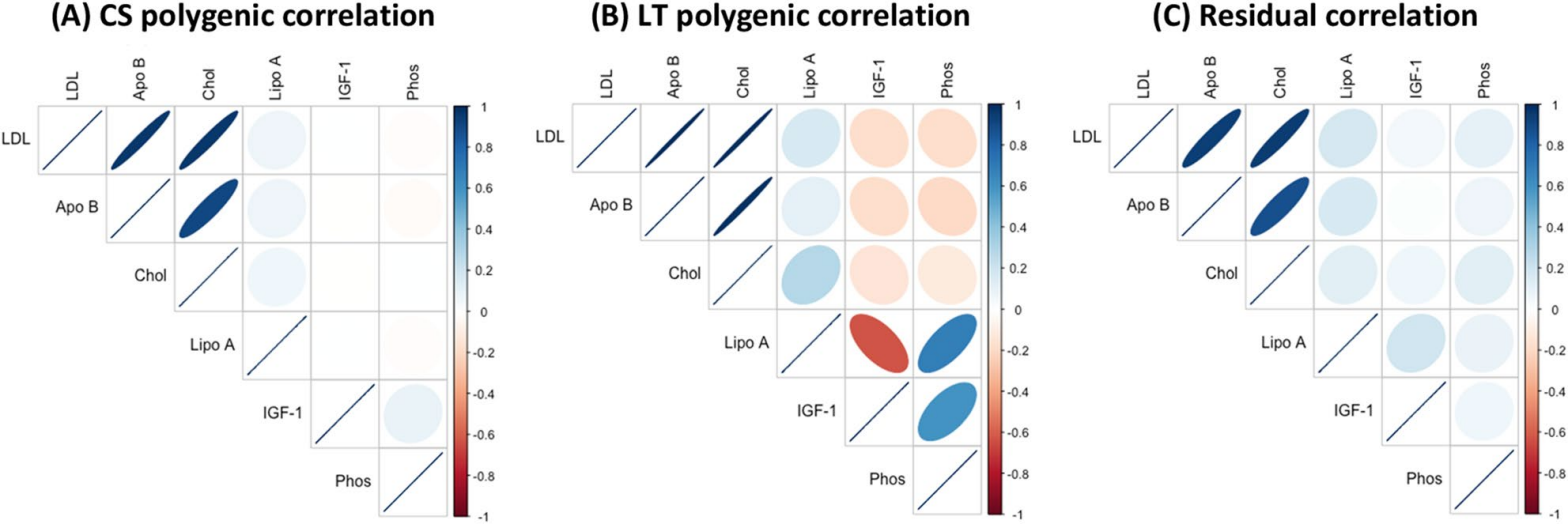
Cross-sectional genetic (**A**), longitudinal genetic (**B**), and residual (**C**) correlation for a cluster of biomarker traits. Genetic and residual correlations were positive and strong among LDL direct, Apolipoprotein B and Cholesterol. In the correlation plots above, thin lines reflect strong correlation and thick lines toward oval and circular shapes indicate weaker correlations toward 0.

Similarly, genetic and residual correlations were positive and strong among LDL direct, Apolipoprotein B and Cholesterol of cluster-4 (Fig. 10). Results indicate that significant pleiotropy exists for variants associated with the levels of these biomarkers as well as their progression. The traits also share a large common environmental component as indicated by the strong residual correlation.

#### Longitudinal GWAS

Results of longitudinal genome-wide association scans performed on biomarker phenotypes as 4 multiple-trait longitudinal runs using GAMUT, 28 single-trait longitudinal runs using GALLOP, and 28 conventional runs using conventional GWAS on biomarker rates of change between first and second measurements were presented in Table 2. LD clumping was performed on the outcomes of the 3 approaches studied using r^2^ of 0.4, a window of 500 kb, and a p-value threshold of 5e-8. Numbers of significant SNPs in the table were taken from the number of clumps found across the genome and summed for all biomarkers.

**Table 2:** Number of significant (p-value < 5e-8) variants associated with cross-sectional (CS) and longitudinal (LT) biomarker traits using multiple-trait and single-trait longitudinal GWAS as well as a conventional rate of change GWAS.

Field ID	Cluster	Biomarker trait	GAMUT^1^		GALLOP^2^		Conventional GWAS^3^
			CS	LT	CS	LT	Rate of change
30890	1	Vitamin D	11	0	10	0	1
30630		Apolipoprotein A	20	0	11	0	0
30760		HDL cholesterol	23	1	23	0	0
30660		Direct bilirubin	57	40	18	0	9
30840		Total bilirubin	63	175	57	0	11
30830		SHBG	26	0	43	0	1
30850		Testosterone	11	0	25	0	0
30740	2	Glucose	5	0	41	0	0
30750		Glycated hemoglobin (HbA1c)	8	0	14	2	0
30880		Urate	31	0	13	0	4
30710		C-reactive protein	24	0	1	0	0
30870		Triglycerides	19	27	2	0	1
30670		Urea	1	0	24	0	0
30700		Creatinine	3	0	19	27	0
30720		Cystatin C	20	1	3	0	0
30610		Alkaline phosphatase	41	0	4	0	1
30730		Gamma glutamyl transferase	14	2	20	1	0
30620		Alanine aminotransferase	2	1	6	0	1
30650		Aspartate aminotransferase	12	0	31	0	1
30860	3	Total protein	5	0	4	0	0
30600		Albumin	2	0	5	0	0
30680		Calcium	4	0	2	0	0
30770	4	IGF-1	6	0	49	0	0
30810		Phosphate	3	0	27	0	1
30790		Lipoprotein A	50	0	34	0	1
30690		Cholesterol	17	0	3	0	1
30640		Apolipoprotein B	35	1	6	0	3
30780		LDL direct	26	0	18	0	1
Total significant associations			539	248	513	30	37

^1^Genome-wide associations for multiple longitudinal traits (1 joint analysis per cluster).

^2^Single-trait longitudinal genome-wide association studies.

^3^Conventional genome-wide association studies on rates of change for each trait.

**Figure 11:**
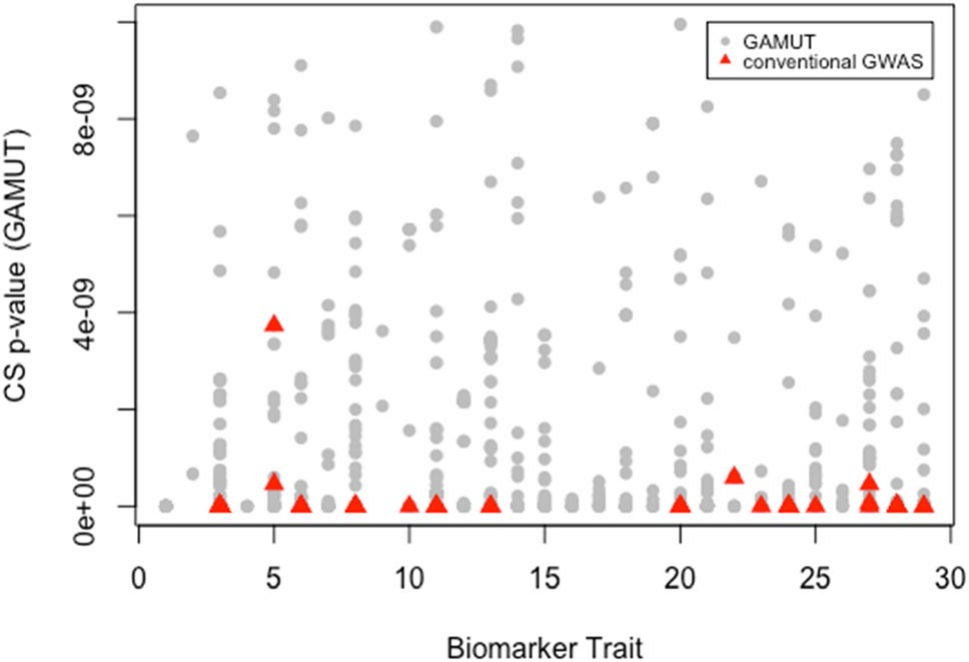
Cross-sectional p-values of all biomarker traits (1532 variants before LD clumping). Significant variants from conventional rate of change GWAS (138 variants before LD clumping) indicated with red triangles. Only the most significant cross-sectional variants were captured by conventional GWAS on rates of change.

Collectively, GAMUT resulted in a total of 539 significant cross-sectional and 248 longitudinal associations, whereas the single-trait longitudinal GWAS resulted in 513 cross-sectional and 30 longitudinal associations. The conventional GWAS on rates of change yielded 37 significant longitudinal associations across the 28 biomarker phenotypes. Agreement between GAMUT and GALLOP was consistent, i.e. 98 of cross-sectional and 90% of longitudinal variants identified by GALLOP were a subset of those identified by GAMUT. There was little overlap between conventional GWAS and the other two methods. All variants identified by rate of change GWAS were observed as cross-sectional associations in the other two approaches. For example, the 9 and 11 variants associated with rate of change in direct and total bilirubin were, in fact, identified as having cross-sectional, but not longitudinal associations with these traits using GALLOP and GAMUT. Additionally, variants identified by rate of change GWAS were generally the most significant cross-sectional associations uncovered by the other two approaches (Fig. 11). This suggests that the approach of fitting rates of change is not an effective method for identifying true longitudinal associations. The additional associations revealed by GALLOP and GAMUT highlight the effectiveness of these approaches in identifying variant associations with both trait level and changes in traits over time. Independent variants revealed by GAMUT, GALLOP, and conventional GWAS on rates of change are provided in Supplementary Tables 1, 2, and 3, respectively.

**Figure 12:**
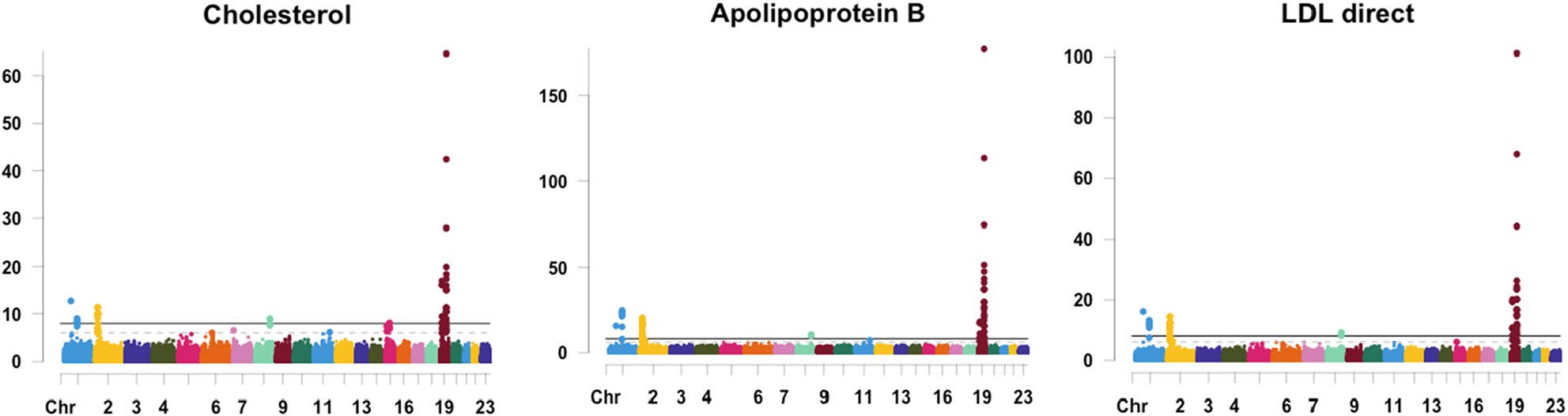
Manhattan plots of cross-sectional variants for 3 genetically correlated traits analyzed jointly using multiple-trait longitudinal GWAS.

Cholesterol, Apolipoprotein B, and LDL direct were identified as genetically correlated traits based on their polygenic variance components (Fig. 10). Log-transformed p-values of the cross-sectional effects of the three traits are shown in Fig. 12. Notice the great similarity between the traits in terms of signal chromosomal location. Number of independent pleotropic variants shared between cholesterol and Apolipoprotein B, cholesterol and LDL direct, and Apolipoprotein B and LDL direct were 15, 16, and 21, respectively.

Cross-sectional effects were well powered and consistent with the biomarker GWAS literature (e.g. Ref.^15^). Longitudinal or progression effects on the other hand were more difficult to validate but still provided multiple clinical insights. In particular, a variant on chromosome 15 (rs117268014), captured only by GAMUT as cross-sectionally and longitudinally associated with both direct and total bilirubin. The variant is in the vicinity of Gremlin1 gene (GREM1) and shown by QTLbase^16^ to be a blood expression QTL for Rho GTPase activating protein 11A (ARHGAP11A), a gene positioned near GREM1 and a methylation QTL for Eukaryotic Translation Initiation Factor 5A2 (EIF5A2), a gene whose overexpression correlates with multiple cancers including colorectal, gastric, and esophageal cancers. Overexpression of the gene was reported to correlate with cancer progression and poor survival^17^. Note also that the role of GREM1 itself is well established in the onset and prognosis of colorectal and gastric cancers. In particular its role in promoting colorectal cancer cell metastasis, motility, and invasion^18^.

### Longitudinal GWAS for triglycerides on CAD-diagnosed patients vs. a random sample

**Figure 13:**
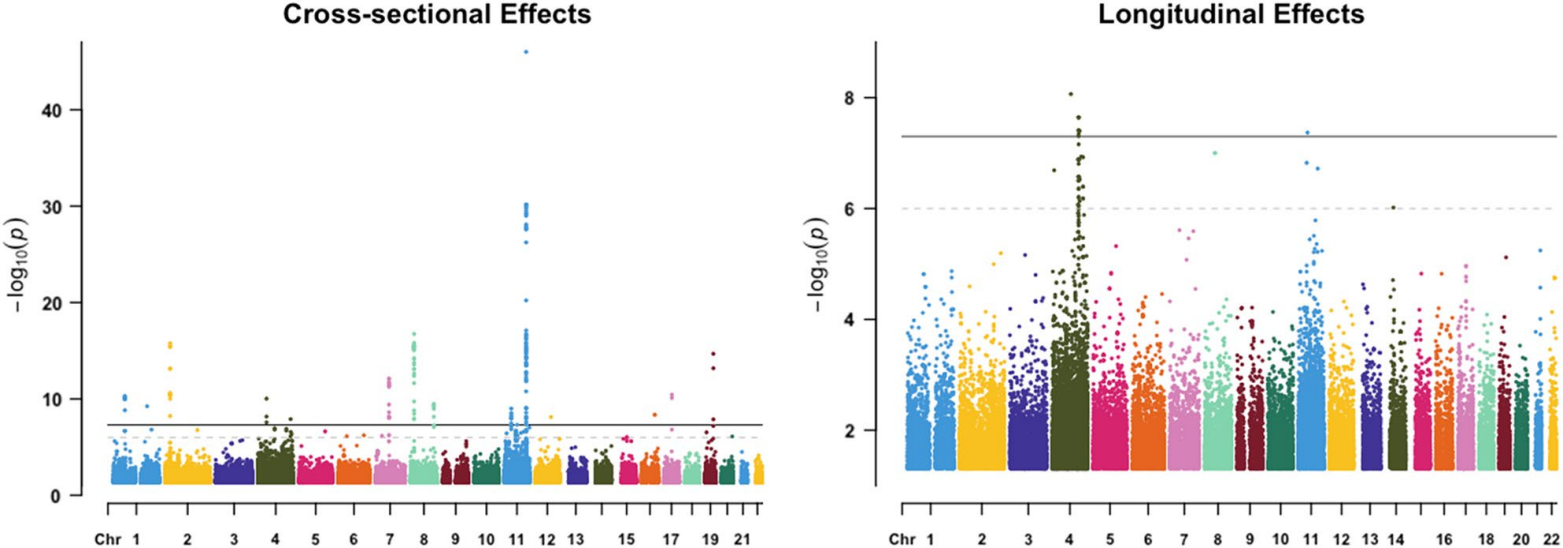
Manhattan plots of cross-sectional and longitudinal variants for primary care triglycerides with 3 to 35 repeated measures on CAD-diagnosed patients. Multiple genome-wide significant hits were identified for the two effect types.

**Figure 14:**
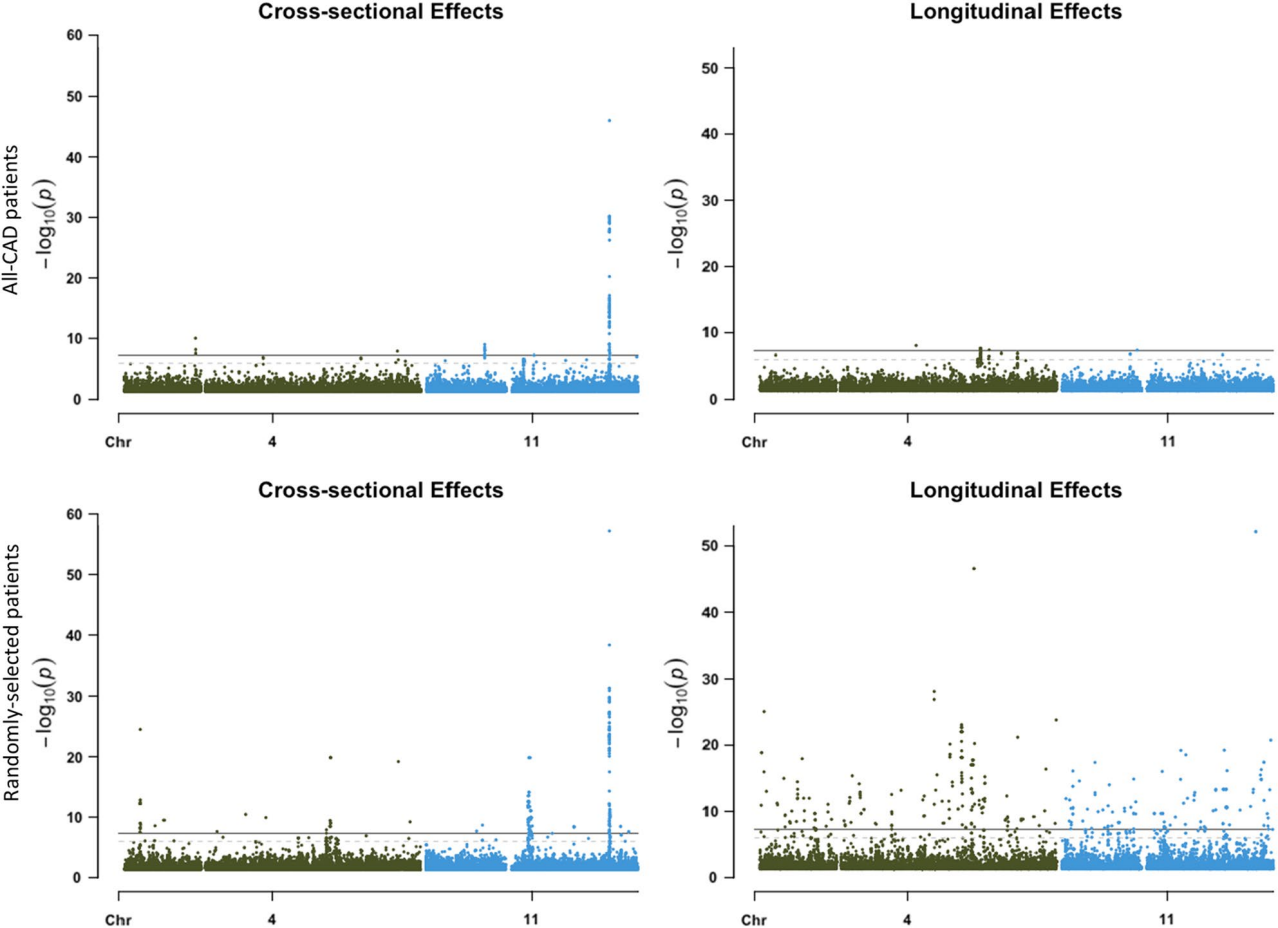
Manhattan plots of cross-sectional and longitudinal variants on chromosomes 4 and 11 for primary care triglycerides with 3 to 35 repeated measures on CAD-diagnosed patients on the top 2 panels vs. a randomly selected sample on the bottom panels. The homogeneous sample with CAD did not show as much inflation as with the random sample.

Unlike the single trait outcomes across the random UK Biobank sample with 2 and 0 cross-sectional and longitudinal genetic signals in Table 2, limiting the longitudinal GWAS to CAD-diagnosed patients with many more repeated triglyceride measures through time resulted in the identification of multiple genome-wide significant cross-sectional and longitudinal signals as can be seen from the Manhattan plots of Fig. 13. The CAD-diagnosed patients were sampled across time and not restricted to those diagnosed before blood samples were taken for each biomarker measure. When using a random group across all diseases, inflation was observed, particularly with longitudinal effects, as can be seen from the bottom 2 panels of Fig. 14. The multiple signals identified compared with the blood biomarkers study of Table 2 highlights the importance of utilizing many repeated measures per individual to increase power. To control for inflation when a mixture of healthy individuals and patients are included in the same longitudinal GWAS for a disease-related trait, we recommend adding a blocking fixed factor in the model to adjust for comorbidity. Given that a group restricted to CAD patients who were diagnosed at any point in their lifetimes reduced noise in longitudinal GWAS signals for triglyceride levels, a polygenic risk score for CAD, in such case, could serve as a stratifying factor to include in longitudinal GWAS models to enhance outcomes and control for inflation in the absence of disease diagnoses data.

## Discussion

In the current study, an efficient and powerful approach to identify genetic variants associated with cross-sectional and longitudinal effects was developed. The approach capitalizes on the availability of broad phenotyping, in which individuals are assessed for multiple clinical traits, as well as deep phenotyping, characterized by multiple measurements of these traits over time. GAMUT successfully modeled both the multiple-trait and the longitudinal aspects of the data while maximizing computational efficiency, outperforming single-trait longitudinal analysis when up to 16 traits were examined. It is imperative to emphasize that a multivariate analysis is inherently more computationally costly than the cumulative cost of the equivalent univariate analyses. The computational advantage of our multivariate approach up to 16 traits is explained by, first, the algorithmic differences between GAMUT and GALLOP in transforming the major random effects matrix of the mixed model equations into an identity, and second, having to build and transform the coefficient matrix only once for all traits. The approach is particularly useful for joint analysis of strongly correlated traits measured in real-world data collected over time. While handling missing values in longitudinal data can be cumbersome, GAMUT provides a framework in which missing records in one or more traits are implicitly estimated based on genetic and environmental covariances with the traits that have more complete data.

Because longitudinal effects are likely to be much smaller than cross-sectional effects as seen in the UK Biobank blood biomarker data, sample sizes required to detect genetic variants associated with disease progression are expected to be manyfold greater than what is required for cross-sectional disease association, as was shown in our simulation. In order for the current approach to be useful, it is essential to have sufficient data at multiple time points per individual, e.g. primary care data or repeated measures in extended large-scale clinical trials. In addition, the UK Biobank continues to assess participants over two time points for thousands of traits such as the repeat MRI imaging study of brain, heart, and abdomen. Although our simulations suggest that multiple-trait approaches offer limited advantages over single-trait analysis in the presence of complete data with no missing observations, such a scenario is unlikely to exist in either observational real-world data collection or prospective clinical trials.

In the current Biomarker analysis, longitudinal variant associations above genome-wide significance suffered from inflation for 3 possible reasons. First, longitudinal effects were orders of magnitude smaller than their cross-sectional counterparts; second, the number of individuals with repeated measurements was limited, and finally time points within individuals were ≤ 2 visits in the current UK Biobank phenotypic data. Inflation was shown to be controlled when a sample of participants, similar in their clinical history and with many more repeated measures, was extracted from the UK biobank primary care data and utilized in longitudinal GWAS.

In this paper, we demonstrate that GAMUT is a computationally efficient framework that accounts for genetic and environmental co-variability in multiple longitudinally assessed quantitative traits. Although univariate modeling approaches have been successfully used to identify important disease associations, they also miss novel insights as they fail to leverage the correlation among pathophysiological processes that lead to disease onset or progression. We have shown this to be the case in an analysis of real-world data from the UK Biobank, where multiple cross-sectional and longitudinal insights would have remained undetected using other standard statistical methods and assert that our approach has broad applicability to large-scale collections of data available in global biobanks and prospective clinical studies today.

### Supplementary Information

Supplementary Tables.

## Data Availability

Blood biomarker data were part of the UK Biobank and can be shared with a research agreement. Underlying data is available for researchers after a material transfer agreement and by following data access procedures at https://www.ukbiobank.ac.uk/enable-your-research/apply-for-access. In addition, genome-wide significant associations data is available in supplementary material.
